# Effect of interpregnancy interval on adverse pregnancy outcomes in northern Tanzania: a registry-based retrospective cohort study

**DOI:** 10.1186/s12884-016-0929-5

**Published:** 2016-06-07

**Authors:** Michael J. Mahande, Joseph Obure

**Affiliations:** Institute of Public Health, Department of Epidemiology & Biostatistics, Kilimanjaro Christian Medical University College, Moshi, Tanzania; Department of Obstetrics and Gynaecology, Kilimanjaro Christian Medical Centre, Moshi, Tanzania

**Keywords:** Interpregnancy interval, Subsequent adverse pregnancy outcomes

## Abstract

**Background:**

Both short and long interpregnancy intervals have been associated with an increased risk of adverse pregnancy outcomes. There is limited information about the impact of interpregnancy interval on pregnancy (IPI) outcomes in Tanzania. The objective of this study was to assess the effect of IPI on adverse pregnancy outcomes.

**Methods:**

We performed a retrospective cohort study using maternally-linked data from Kilimanjaro Christian Medical Centre (KCMC) birth registry. A total of 17,030 singlet births from women who delivered singleton infant at KCMC from 2000 to 2010 were studied. Women with multi-fetal gestations and those who were referred from rural areas for various medical reasons were excluded. Outcome variables were preterm birth, low birth weight infants and perinatal death. A multiple logistic regression was used to assess the association between IPI and pregnancy outcomes.

**Results:**

The median IPI was 36 months. Compared with IPIs of 24–36 months (referent group), short interpregnancy intervals (<24 months) was associated with preterm delivery (OR 1 · 52; 95 % CI 1.31–1.74); low birth weight (OR 1 · 61; 95 % CI 1 · 34–1.72) and perinatal death, (OR 1 · 63; 95 % CI 1.22–1.91). The IPI of 37–59 months or longer were also associated with higher risks of preterm birth and low birth weight, but not with perinatal death.

**Conclusions:**

Our study confirmed that both short and long IPI are independent risk factors for adverse pregnancy outcomes. These finding emphasize the importance of providing support for family planning programs which will support optimal IPI and improve pregnancy outcomes.

## Background

Inter-pregnancy interval (IPI) is defined as the time lapsed between two consecutive pregnancies [1]. Although some investigators in Brazil have considered a short IPI when it is less than 6 months and long IPI when it is more than 5 years; IPI of 24 months is considered to be optimal [1, 2]. Poorly timed pregnancies increase health risks for both mother and infant while optimal interpregnancy interval (IPI) is an important determinant of maternal health and pregnancy outcomes [1, 2]. However, it is noteworthy that categorization of IPI differs a bit between studies, making it difficult comparing different study findings. [1–6].

Previous studies in low and high income countries have shown that both short and long IPIs are associated with adverse maternal, perinatal and infant outcomes [1–3]. Particularly, short IPI is linked with greater risks of perinatal, infant and child mortality, preterm birth, low birth weight and fetal growth restriction [2, 4–6]. Furthermore, short IPI has been associated with congenital malformation, maternal anemia, premature rupture of membranes, abruption placenta, placenta previa and uterine rupture particularly in women with previous cesarean section delivery attempting vaginal delivery [1, 4, 7–10]. Some adverse perinatal outcome such as preterm birth and low birth weight are associated with increased morbidity and mortality for newborn and infant [11, 12]. In addition, babies born prematurely or with low birth are at a higher risk of long term complications [13, 14]. On the other hand, long IPI has been associated with increased risks of preterm birth, low birth weight, labour dystocia, preeclampsia and eclampsia [1, 15].

The demographic and Health Surveys (DHS) report from developing countries has indicated that short IPI of six months or less is associated with increased risk of low birth weight, fetal growth restriction, early neonatal, infant and child mortality as compared to IPI of 36 months or more [16, 17]. Moreover, the analysis of DHS data from five sub-Saharan Africa countries including Tanzania showed that pregnancies occurring after IPI of less than 15 months are more likely to end in perinatal deaths as compared to pregnancies that occurred after long inter-pregnancy intervals [18].

Findings from a community-based study in Tanzania revealed that 50 % of the pregnancies follow an optimal interpregnancy interval recommended by the World Health Organization (WHO) of 24 months before attempting next pregnancy. This interval in addition to 9 months of pregnancy period add up to a minimum length of 33 months between two consecutive live births and hence associated with reduced risk of adverse maternal, perinatal and infant outcomes [19]. However, it is important not to confuse IPI which is the focus of this paper with a birth interval which is defined as the time between two consecutive live births. The lack of adherence to an optimal IPI as recommended by the WHO may be attributed to low contraceptive prevalence rate in Tanzania [20]. According to the Tanzania Demographic and Health Survey report in 2010, the contraceptive prevalence among women aged 15–49 years in Tanzania was estimated to be 34.4 % [20]. Exclusive breast feeding for six month is one of the natural birth control methods as it delays a woman’s return to ovulation. Exclusive breastfeeding improves both infant survival and lengthens the interval between pregnancies due to lactational amenorrhea. However the risk of conception increases as the breastfeeding decreases or when the menstruation resumes. This indicates that mothers should not wait to start using contraceptives until the return of their menstrual period to prevent unwanted pregnancies and enabling mothers to adhere with the recommended optimal birth spacing. However, only 50 % of the women in Tanzania practice exclusive breast feeding up to six months [20]. The lower prevalence for contraceptive use and exclusive breastfeeding contribute to the reported poor timing of pregnancy among Tanzania women [19]. This calls for the efforts to increase access and utilization of contraceptive services among women of reproductive age.

There are, however, limited studies conducted on the impact of IPI on pregnancy outcomes in sub-Saharan Africa particularly in Tanzania. Lack of population-based linked data on women’s reproductive history is striking. In this paper we assessed the effect of IPI on adverse pregnancy outcomes (preterm birth, low birth weight and perinatal death) using hospital based maternally-linked registry data in northern Tanzania.

## Methods

We conducted a retrospective cohort study using maternally-linked data from Kilimanjaro Christian Medical Centre (KCMC) medical birth registry for women who were recorded with at least one singleton infant birth at KCMC during the year 2000 to 2010.

KCMC is one of the four zonal referral hospitals located in Moshi urban district, Kilimanjaro region in the northern Tanzania. The hospital receives patients from the nearby communities in the region and referred cases from the neighboring regions. Majority of the study population were women who had at least two singleton deliveries at KCMC. The description of the KCMC medical birth registry has been presented in previous study [21].

We used women reproductive history data that were collected from all women who delivered at the Department of Obstetrics and Gynecology Unit. The data were collected within 24 h after delivery, or as soon as mothers had recovered in case of complicated deliveries. On daily basis, trained nurse midwife conducts interviews using a validated and standardized questionnaire (which is a standard practice). Information that are collected during the interviews include: 1) paternal and maternal demographic characteristics 2) women reproductive history 3) Condition of the mother before pregnancy, during pregnancy and after pregnancy and attendance to antenatal care clinics) 4) labor management 5) maternal complications during pregnancy/delivery and puerperium 6) and neonatal outcomes. In addition, data from the patient’s case file were extracted to supplement the missing information. During the interview, women were also requested to provide reproductive information for deliveries that occurred outside KCMC during the study period. Verbal consent was sought from each individual mother prior to the interview. Data were entered in a computerized data base system at the birth registry.

We restricted our study to women who were recorded with least two or more singleton births (live birth or fetal death at ≥28 weeks of gestation) during the study period. This contributed to 17,030 singleton births. We excluded births for women who had multi-fetal gestations and those who were referred from rural areas for various medical reasons.

In order to keep track of each woman’s pregnancy outcome a unique hospital identification number was used to link mother’s with their subsequent offspring. However, we were not able to link data for mothers who experienced miscarriage/abortion as KCMC do not capture their information.

The outcome variables for this study were preterm birth, low birth weight and perinatal death in the subsequent pregnancy. The main predictor variable was IPIs categories between the two successive pregnancies. In this study the IPI was defined as period between the beginning of first pregnancy and the date of the last menstrual period of the subsequent pregnancy. The IPI was originally measured in days, and then converted in months. In this study the IPI was categorized as 24–36 months (as a reference group/optimal IPI); less than 24 months (considered as short IPI), 37–59 months (moderate IPI), and ≥60 months (as long IPI).

Preterm delivery was defined as delivery of a live infant before 37 weeks of gestation. Gestational age at birth was estimated as the interval in completed weeks from the last normal menstrual period (LMP) to the child’s date of birth. Perinatal deaths refer to fetal deaths and live births with only brief survival (days or weeks). It comprises, fetal deaths with a stated or presumed period of gestation of 28 weeks or more (stillbirth) and infant deaths that occur at less than 7 days of age (early neonatal death). Low birth weight was defined as an infant birthweight less than 2500 g.

The following covariates were considered to be confounders; maternal age, maternal marital status, maternal educational status, maternal occupation, parity, area of residence, number of antenatal care visits (ANC), use of family planning methods and use of alcohol during pregnancy.

### Statistical analysis

Data were analyzed using statistical package for social science (SPSS) version 18.0, Stata version 12.0, and R version 2.15.2. A chi square (*χ*^2^) test was used to compare various maternal and fetal characteristics across different IPI categories. The rates of adverse pregnancy outcomes were estimated for each IPI category. We estimated the effect of IPI on preterm birth, low birth weight and perinatal death in the subsequent pregnancy independent of the confounder using multiple logistic regression models. We used indicator variables to represent the IPIs categories (<24, 24–36, 37–59, and ≥60 months). We also used robust variance estimation to take into account for repeated observations or correlation between siblings of the same biological mother. A p-value of less than 0.05 was considered to be significant.

In order to determine whether a dose response existed between IPI and the studied pregnancy outcomes, we repeated the analysis using Generalized Additive Models (GAMs) [22], treating IPI as a continuous variable as this may also minimize underestimation of the risk of short IPI as opposed to categorization of IPI duration. In this analysis, the OR was calculated using the population average as the reference. Calculations were performed using the R package Mixed GAM Computation Vehicle (mgcv), adjusting for the same confounders as mentioned above. Since previous pregnancy outcomes are associated with IPI and adverse pregnancy outcomes in the subsequent pregnancy, we also tested the influence of the previous pregnancy outcomes studied on IPI to ascertain if they were associated with IPI. For example, preterm birth delivery may lead to short IPI in the subsequent pregnancy. Similarly, women who experience perinatal loss tends to immediately go for the next pregnancy after a short period to replace the pregnancy loss. These outcomes also tend to recur between pregnancies.

## Results

### Characteristics of the study participants

In total 17,030 singleton births were included in the study. Short and long IPIs occurred in, 19.42 % and 14.31 % of 17,030 singleton births while 32.34 % and 33.92 % of births occurred after moderate and long IPIs respectively. Median IPI was 36 months. Women with shorter IPIs were more likely to be young (<19 years), employed, and with high education attainment than referent group. Women with long IPIs were more likely to have high maternal age (≥35 years), being single and obese (Table 1).

**Table 1:** Maternal characteristics in relation to interpregnancy interval (*n* = 17,030 births)

Characteristics	Interpregnancy interval (month)			
	<24 (*n* = 3309)	24–36 (*n* = 5774)	37–59 (*n* = 5509)	≥60 (*n* = 2442)
Maternal age (years)				
≤ 19	113 (3.41)	111 (1.92)	20 (0.36)	3 (0.12)
20–34	2750 (83.11)	4826 (83.58)	4384 (79.57)	1503 (61.55)
≥ 35	446 (13.48)	837 (14.49)	101 (1.83)	936 (38.33)
Mothers education (years)				
< 12	2120 (64.06)	4073 (70.54)	3958 (71.84)	1757 (71.95)
≥ 12	1189 (35.94)	1701 (29.46)	1547 (28.16)	685 (28.05)
Marital status				
Single	148 (4.47)	257 (4.45)	267 (4.85)	239 (9.79)
Married	3161 (95.53)	5517 (95.55)	5238 (95.15)	2203 (90.21)
Parity^a^				
1	1905 (57.57)	2839 (49.17)	2480 (45.02)	1006 (41.19)
2	666 (20.12)	1466 (25.39)	1560 (28.32)	744 (30.47)
≥3	659 (22.31)	1333 (25.44)	1320 (26.66)	612 (28.34)
Number of ANC visits^a^				
< 4	1127 (34.06)	2018 (34.95)	1638 (29.73)	786 (32.19)
≥ 4	2182 (65.94)	3756 (65.05)	3867 (70.27)	1656 (67.81)
Mothers area of residence				
Rural	1317 (39.80)	2582 (44.72)	2143 (38.89)	896 (36.69)
Urban	1992 (60.20)	3192 (55.28)	3362 (61.11)	1546 (63.31)
Occupation^a^				
Unemployed	2611 (78.91)	4841 (83.84)	4740 (86.04)	2078 (85.09)
Employed	687 (20.09)	904 (15.66)	739 (13.96)	349 (14.91)
Alcohol use during pregnancy	1133 (34.24)	2268 (39.28)	2372 (43.07)	1035 (42.38)
Use of family planning methods	933 (28.19)	460 (7.96)	248 (4.50)	183 (7.49)
Pregnancy BMI (kg/m^2^)^a^				
Underweight (<18.5)	5 (0.15)	18 (0.31)	13 (0.24)	7 (0.29)
Normal (18.5–24.9)	1647 (49.77)	2901 (50.24)	2693 (48.88)	1064 (43.57)
Overweight (25–29.9)	827 (24.99)	1497 (25.93)	1377 (24.88)	609 (24.94)
Obese (≥30)	427 (25.09)	607 (23.52)	737 (26.0)	43 (31.20)

Values in brackets are percentage of births in each IPI category

^a^Numbers do not add to total because of missing data

### Rates of adverse pregnancy outcomes in relation to IPIs

Overall, both short and long IPI were associated with increased rates of preterm birth and low birth weight. Compared with the reference group, short IPI was associated with high rates of preterm birth (10.51 % vs.12.57 %), low birth weight (8.5 % vs.11.97 %), and perinatal death (2.92 % vs. 4.14 %) whereas longer IPI was associated with high rates preterm birth (10.16 %), and low birth weight infants (8.64 %). The rate of perinatal death decreased overtime as the IPI increased (Table 2).

**Table 2:** Rates of adverse pregnancy outcomes in subsequent pregnancy by interpregnancy intervals (*n* = 17,030 births)

	Interpregancy interval (years)			
	<24	24–36	37–59	≥60
Outcome	(*n* = 3309)	(*n* = 5774)	(*n* = 5509)	(*n* = 2442)
Preterm birth	416 (12.57)	554 (9.59)	489 (8.88)	248 (10.16)
Low birthweight	396 (11.97)	489 (8.47)	472 (8.57)	211 (8.64)
Perinatal death	137 (4.14)	170 (2.94)	171 (3.10)	79 (3.24)

### Multivariate analysis

Results of the multivariate logistic regression models after adjusting for confounders are presented in Table 3. Compared with the reference group (IPI of 24–36 moths), short IPI was significantly associated with increased risk of preterm birth (adjusted OR, 1.52; 95 % CI: 1.31–1.74), low birthweight infant (adjusted OR, 1.61; 95 % CI: 1.34–1.72), and perinatal death (adjusted OR, 1.63; 95 % CI: 1.22–1.91). On the other hand, infants conceived after longer IPI had increased odds of preterm birth and low birth weight (adjusted OR, 1.13; 95 % CI: 1.02–1.24; and 1.11; 95 % CI: 1.04–1.2) respectively. Furthermore, women who conceived within a moderate IPI had slight elevated risks for preterm birth and low birth weight. Conversely, both moderate and longer IPIs were associated with decreased odds of perinatal death.

**Table 3:** Adjusted odds ratios (95 % CI) for the association between interpregnancy interval and adverse pregnancy outcomes

	Interpregancy interval (month)			
Outcome	<24	24–36 (reference)	37–59	≥60
Preterm birth	1.52 (1.31–1.74)	1.00	1.02 (0.79–1.21)	1.13 (1.02–1.24)
LBW	1.61 (1.34–1.72)	1.00	1.09 (0.97–1.16)	1.11 (1.04–1.21)
Perinatal death	1.63 (1.22–1.91)	1.00	1.06 (0.86–1.32)	1.07 (0.87–1.32)

**Fig. 1:**
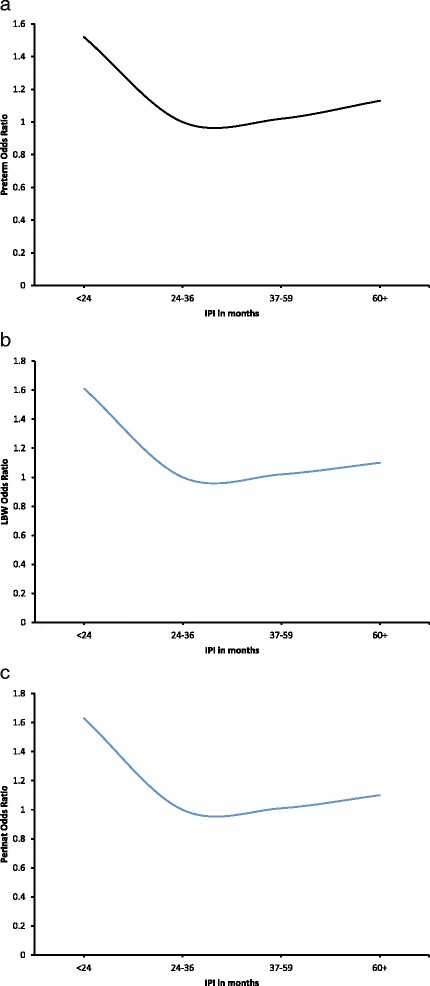
**a** Relationship between Interpregnancy interval and preterm birth. **b** relationship between interpregnancy interval and low birth weight. **c** relationship between interpregnancy interval and perinatal death

We repeated the analysis using Generalized Additive Models (GAMs), treating IPI as a continuous variable to test for non-linearity. We wanted to determine whether there was a dose response relationship between IPI and outcomes of interest. The IPI was treated as a continuous variable in the model. This analysis confirmed the above trends on relationship between IPI and risk of the pregnancy outcomes studied (Fig. 1), indicating that our results were not biased by categorization of the IPI. Although the risk of perinatal death decreased over time, it reached a certain point the risk of perinatal death slowly increased linearly as IPI increases.

In the sub-analysis, we assessed the association between IPI and previous pregnancy outcomes (preterm birth, low birth weight and perinatal death) controlling for the same confounders (Table 2). Since the length of IPI can be influenced by some of these outcomes such as preterm birth and perinatal death, we wanted to ascertain whether the previous pregnancy outcome was associated with IPI in the index pregnancy. These factors were only significantly associated with short IPI; and when we included previous pregnancy outcome in the models, the estimated odds ratios for perinatal death remained unchanged (data not shown).

## Discussion

In this study both short and long IPIs were associated with higher risks of preterm birth, and low birth weight. We found that infants born 24–36 months after the previous birth had the lowest risk of preterm birth, low birth and perinatal death as compared to those who were born after shorter or longer IPIs. In addition, short IPI was associated with an increased risk of perinatal death, but the risk of perinatal death decreased with an increase in IPI. We also noted that the risk of perinatal death goes up with long IPI.

The association between adverse perinatal outcomes and both short and long IPIs have been previously reported [23–25]. A study in Israeli by Grisaru-Granovsky and colleagues [4], found that women who conceived at either shorter (less than 6 months), or longer (60 months) IPIs had greater risk of preterm birth. Adam and colleagues [26] in Sudan also found that women who conceived after IPI of less than 18 months were more likely to have preterm birth and low birth weight infants compared with those who conceived after of 18–30 months. Consistent to our study, both short and long IPIs were independently associated with higher risks of preterm and low birth weight. Furthermore, short IPI was associated with an increased risk of perinatal death, while long IPI was associated with lowest risk of perinatal death. The high risk of perinatal death among infants who were born after short IPI may be explained by the effect of prematurity or low birth weight because infants who are born premature or with low birth weight have greater risk of perinatal death as compared to infants who are born at term or with normal birth weight [11, 12, 21]. In addition, the high risk of perinatal death after short IPI could be explained by recurrence of causative factors of perinatal death between pregnancies. We also noted that the risk of perinatal death goes up as the IPI increases. This probably may be explained by the effect of increase in maternal age, as older women have high risks of adverse outcomes or increased pregnancy complications such as preeclampsia which is also related to both perinatal death and long IPI.

Klerman and coworkers [27] reported that short IPI has high impact on pregnancy outcomes among poor women in low income countries, especially those who experience poor nutrition, physical and social stresses as compared to women in the high income countries. Our findings support this evidence as short IPI was significantly associated with increased risks of preterm birth, low birth weight and perinatal death, all these factors contributes to high neonatal and child mortality in low income countries [16–18]. Our finding suggest for urgent need for intervention to promote women to optimize IPI of 24–36 moths to reduce these adverse perinatal death in order to achieve Millennium development goal for improved child survival by 2015 and beyond.

The relationship between short IPI and adverse perinatal outcomes such as preterm birth, low birth weight, fetal growth restriction and infant mortality has been attributed to maternal nutrition depletion and postpartum stress [28, 29]. Maternal nutrient depletion is defined as a negative change in maternal nutritional status during a reproductive cycle which may pose biological competition between mother and the growing fetal [28]. This could be the possible explanations for increased risks of preterm birth and low birth among women with short IPI in the present study. It is believed that short IPI do not provide a mother with sufficient time to recover from the nutritional burden and stress of the previous pregnancy. This leads to maternal nutrition depletion which compromises the mother’s ability to support fetal growth and development which in turn increases the risks of preterm birth, growth restriction as well as maternal morbidity and mortality in the subsequent pregnancy.

The previous authors also found that women with short IPI enter in their subsequent pregnancy with low nutritional reserves [28, 29]. Therefore, inadequate maternal nutrients supply creates nutritional imbalance between the mothers and fetal which leads to biological competition between mother and fetal leading to adverse perinatal outcomes. On the other hand, short IPI is associated with maternal iron and folic acid depletion which is also linked with increased risks of preterm birth, low birth weight and growth restriction [28]. This may also be the reason for increased risk of preterm birth and low birth weight in our study. Other possible explanations for increased risk of preterm birth and low birth weight after short IPI could be due to the recurrence of these outcomes, preeclampsia or interaction of prior pregnancy outcomes and IPI [30, 31]. As shown in sub-analysis, previous pregnancy outcome to some extent has an influence on IPI which may also explain the observed relationship. However, it is possible that the increased risk of low birth weight infants may be a result of preterm delivery or small for gestation age which is main reasons for low birth weight and indirect causes of neonate deaths.

On the other hand, the increased risk of preterm birth and low birth weight after long IPI may partly be due to the coexistent of maternal complications such as preeclampsia, hypertension, obesity and diabetes which are more prevalent as IPI increases. Another hypothesis for the association between long IPI and increased risks of preterm birth and low birth weight could be explained by decline in mother’s physiological and anatomical adaptation of the reproductive system which decline gradually after a long time if a woman does not conceive another fetus. During this time, the mother’s physiological characteristics resemble to those of primigravida [1, 15]. However, the association between adverse perinatal outcomes and long IPI may attributed by factors which cause secondary infertility which tends to increase as the IPI increases [6].

Previous authors found that women who conceive at less than six months are more likely to have preterm birth, low birth weight and perinatal death in their next pregnancy as compared to those who conceived later [32]. Consistent with our study, women who conceived within a shorter IPI were more likely to experience perinatal death in their next pregnancy as compared to those who waited for moderate or longer intervals. This could be explained by the presence of infections, stress or life style in the subsequent pregnancy [16]. In addition, closely spaced infants’ increases the exposure to infection for a newborn from the elder sibling which increases the risk of perinatal death. Long IPI may help a mother to recover from the stress of the previous pregnancy and reduce the risk of infections between siblings.

Furthermore, maternal nutritional depletion and other postpartum related stress increases the risk of perinatal and infant mortality in subsequent pregnancy for closely spaced pregnancies [32]. However, it may also be possible that these women had previous complications such as preeclampsia that recurred in the successive pregnancy. Short IPIs might be attributed to other factors such as poor socioeconomic status and previous perinatal death which are also common among women with short interpregnancy intervals. Alternatively, maternal infections, malaria, iron deficiency anemia, maternal stress or life style compounding to an already nutritionally depleted body may have contributed to these complications.

Our data have a number of limitations which need to be taken into account when interpreting these results. First, since this is a hospital-based study, selective-referral bias may affect our results if high-risk pregnant women and those with complications were overrepresented. In many cases women with previous obstetric problems are more likely to deliver in tertiary facilities in their next pregnancy. However, we excluded from the analysis all women referred from rural area for various medical complications during first and second pregnancies to avoid overestimation of risk to minimize the effect of such bias. Secondly, missing records and inaccurate estimation of gestational age may result in misclassification bias of preterm births. Thirdly, only a small proportion of women had long IPI which may lead to an underestimation of the effect of long IPI on adverse pregnancy outcomes. Fourth, it is important to note that different studies used different cut off points for IPI. Therefore, the optimal IPI might differ a little bit between studies. This pose challenges in comparing results between studies. Finally, we used a unique maternal identification number to link mothers with their sibling. If our matching criteria failed due to women changing their identifications between pregnancies, this may have contributed to loss of follow-up.

To the best of our knowledge, this is the first largest cohort in Tanzania and perhaps in sub Saharan Africa to assess the effect of interpregnancy intervals on subsequent risks of adverse pregnancy outcomes. This study provides critical information to health care providers, which are important for designing programs for optimal birth spacing to improve maternal, newborn and child survival. The use of a unique maternal identification number to link with their sibling minimizes recall bias. The registry data also contains detailed information of the mothers before and after pregnancy which enabled us to adjust for the most important clinical and sociodemographic confounders associated with adverse perinatal outcomes studied. We excluded women who were referred from rural areas for various medical reasons to minimize selection bias that may be attributed to the overrepresentation of the high-risk women.

## Conclusions

Our study confirmed the findings of previous studies that both shorter and longer IPIs are associated with increased risks of adverse pregnancy outcomes. This study provides critical information relevant for improving pregnancy outcomes and fetal survival. Health care providers should identify and counsel women who have recently given birth regarding the effect of IPIs (short or long) and the risk of subsequent pregnancy to optimize the interval for good pregnancy outcomes. These data emphasize the importance of providing support for family planning services which will promote optimal IPI and improve pregnancy outcomes.

## Abbreviations

ANC, Antenatal care; CI, Confidence Interval; DHS, Demographic Health Survey; GAMs, Generalized Addiditive Models; IPI, Interpregnancy interval; KCMC, Kilimanjaro Christian Medical Centre; LMP, Last menstrual period; mgcv, Mixed GAM Computation Vehicle.; OR, Odds ratio; P, Probability; SD, Standard deviation; SPSS, Statistical package for social science; *χ*^2^, Chi-square test
